# Immunosenescence: participation of T lymphocytes and myeloid-derived suppressor cells in aging-related immune response changes

**DOI:** 10.31744/einstein_journal/2019RB4733

**Published:** 2019-04-25

**Authors:** Amanda Soares Alves, Valquiria Bueno

**Affiliations:** 1 Universidade Federal de São Paulo, São Paulo, SP, Brazil.

**Keywords:** Immunosenescence, T-lymphocytes, Inflammation, Myeloid-derived suppressor cells, Aging, Imunosenescência, Linfócitos T, Inflamação, Células supressoras mieloides, Envelhecimento

## Abstract

Healthy aging is partly related to appropriate function of the immune system. As already reported, some changes in this system are observed, including reduced number and repertoire of T cells due to thymic involution, accumulation of memory T cells by chronic infections, homeostatic proliferation compensating for the number of *naïve* T cells, decreased proliferation of T cells against a stimulus, telomere shortening, replicative senescence of the T cells, and inflammaging, besides the accumulation of myeloid-derived suppressor cells. The purpose of this article is to clarify each of these changes, aiming to minimize limitations of immunosenescence. If such associations can be established, these cells may be used as early and less invasive markers of aging-related diseases, as well as to indicate interventions, evaluate the efficacy of interventions and be a tool to achieve longevity with quality of life.

## RESUMO

O envelhecimento saudável está relacionado, pelo menos em parte, com a função adequada do sistema imunológico. Isso porque já foi relatado que, com o envelhecimento, algumas mudanças desse sistema são observadas, como a diminuição da percentagem e do repertório de células T pela involução tímica, acúmulo de células T de memória por infecções crônicas, compensação do número de células T *naïve* por proliferação homeostática, diminuição da capacidade de proliferação das células T frente a um estímulo, encurtamento dos telômeros, senescência replicativa das células T, e *inflammaging*, além do acúmulo de células mieloides supressoras. Este artigo visa esclarecer cada uma das mudanças, mencionadas, com o intuito de buscar meios de minimizar as limitações da imunosenescência. Caso seja possível estabelecer tais relações, essas células podem ser utilizadas como marcadores precoces e pouco invasivos de doenças relacionadas ao envelhecimento, além da possibilidade de serem utilizadas para indicar intervenções, avaliar a eficácia das intervenções e como ferramenta para alcance da longevidade com qualidade de vida.

## INTRODUCTION

According to the World Health Organization (WHO), Brazil will rank sixth in countries with the highest number of elderly individuals by 2025. As already observed in other places around the world, this increase in the elderly population may have a socioeconomic impact in the country, since the aging process comes with associated diseases. Some of the most frequent comorbidities in the seniors are related to changes in the immune system brought by the aging process, called immunosenescense. These alterations lead to increased incidence and severity of infectious diseases, and to insufficient protection after vaccination, which result in a great number of admissions to hospital.^(^ ^1^ ^)^

## IMMUNOSENESCENCE

Immunosenescence was initially defined as a group of changes that occur in the immune response during the aging process, and is a synonym for immunodegeneration. The reason for that is the immune system was believed to collapse with the aging process, considering the increased susceptibility of these individuals to infectious diseases and developing cancer, reduced production of antibodies against specific antigens, increase in autoantibodies, decrease in T-lymphocyte proliferation, in addition to thymic involution.^(^ ^2^ ^,^ ^3^ ^)^ However, immunosenescence is currently defined by some researchers as remodeling of the immune system, suggesting plasticity of the immune system in the aging process. According to these researchers, the aging process does not necessarily bring an inevitable decline of immune functions; what happens is a rearrangement or an adaptation of the immune system to adjust the body that has been exposed to different pathogens throughout life. Depending on how successful that rearrangement or adaptation is, senior individuals can reach longevity with quality of life or, conversely, develop chronic diseases (comorbidities) and/or be often hospitalized due to severe infections.^(^ ^4^ ^,^ ^5^ ^)^

**Figure 1:**
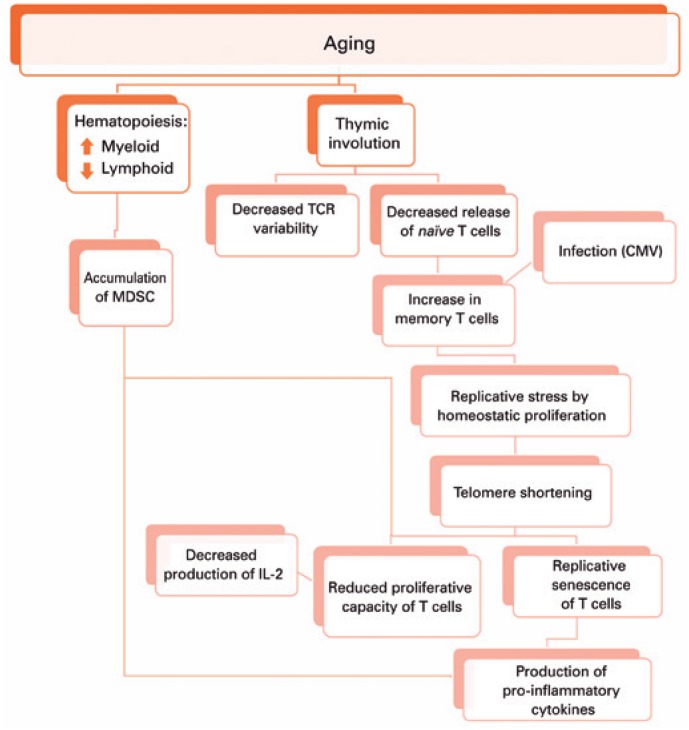
Changes resulting from aging MDSC: myeloid-derived suppressor cells; TCR: T cell receptor; CMV: cytomegalovirus; IL-2: interleukin 2.

This adaptation of the immune system brought by aging seems to result in reduced number and repertoire of T cells due to thymic involution, accumulation of memory T cells from chronic infections, homeostatic proliferation compensating for the number of *naïve* T cells, decreased proliferation capacity of T cells against stimuli, T cell replicative senescence and inflammaging, besides accumulation of myeloid-derived suppressor cells (MDSC)^(^ ^6^ ^)^ (Figure 1).

As we get older, during hematopoiesis in the bone marrow, the myeloid lineage tends to increase, which can favor the accumulation of MDSC. These cells are able to supress T cells proliferation and function, and produce pro-inflammatory cytokines. Moreover, there is thymus involution and replacement of thymic tissue by adipose tissue. Hence, there is reduced T cell receptor (TCR) variability and release of *naïve* T cells. The decreased thymic release of *naïve* cells, together with the immune response against infections throughout life, lead to the accumulation of memory T cells. In elderly individuals, both *naïve* and memory T cells can be maintained thanks to homeostatic proliferation, which shortens the telomeres of these cells, resulting in replicative senescence of T cells that produce pro-inflammatory cytokines, and promote inflammaging. The shortening of telomeres also decreases the proliferation capacity of T cells, which will produce less interleukin-2, further decreasing the proliferation of these cells.

Considering T cells are essential for the adequate response against pathogens and neoplasms, and for protection after vaccination, it seems reasonable that changes in T cells quantity, phenotype, and function play an important role in immunosenescence. Therefore, T cells could be used as biomarkers. Our group focused on the evaluation of T cell phenotype and function and on MDSC, which have been recently associated to aging and whose role in this process is still unclear. Some studies reported an increased percentage of MDSC in tumors and infections, and a possible role in Alzheimer's disease.^(^ ^7^ ^,^ ^8^ ^)^ Below, you will find a description of each mechanism mentioned above, originated by remodeling of the immune system with aging.

## T CELLS

### Thymus

Immunosenescence reduces the recognition of new antigens, which occurs due to decreased TCR variability by thymic involution. This process contributes to increased susceptibility of aged individuals to infectious diseases, or to an ineffective response to vaccination.^(^ ^6^ ^)^ Francisco et al., evaluated data of elderly in São Paulo, and observed that although being vaccinated against influenza virus, the individuals aged over 80 years were more often admitted to hospital due to respiratory diseases, as compared to those aged 60-64 years.^(^ ^9^ ^)^ Some seniors presented a decreased immune response after vaccination; nonetheless, this type of prevention is essential. A survey by Nichol et al., showed in 10 vaccination seasons, there was a drop by 27% in the risk of hospitalization due to influenza virus pneumonia, and a reduction by 48% in the risk of death.^(^ ^10^ ^)^

Thymus involution is also related to a low production of *naïve* T cells in the elderly. In young individuals, the amount of *naïve* T cells and memory T cells is similar. In immunosenescence, there are more memory T cells than *naïve* T cells.^(^ ^11^ ^)^

### Phenotype

In a recent study conducted by our group, we observed there were already some changes at the initial stage of aging (60 to 65 years), including decreased *naïve* T cells and accumulation of terminal differentiated effector memory T cells (T_EMRA_), and such alterations changes were more marked in men.^(^ ^12^ ^)^ Hamann et al., also found a relation between aging and increased number of TCD8^+^ T_EMRA_.^(^ ^13^ ^)^ This memory T cell accumulation in the elderly, as well as the relative reduction in the specificity diversity of TCR recognition could interfere in the process of immune responses to new antigens in those individuals.

Cytomegalovirus (CMV) infection is associated to an increase in effector memory T cells (T_EM_).^(^ ^14^ ^)^ The relative roles that aging and CMV infection play throughout life in the formation of *naïve* TCD4^+^ cells and memory T cells in healthy seniors are not yet clear. However, it was observed that aging is more relevant than CMV seropositivity for decrease in *naïve* T cells throughout time. Nevertheless, the rise in TCD4^+^ T_EM_ and T_EMRA_ cells almost exclusively results from CMV seropositivity. A large proportion of TCD8^+^T_EM_ and T_EMRA_ cells in elderly individuals can also be specific to CMV. Thus, although CMV infection is harmless to young and healthy individuals, it can affect immune dysfunction during aging, which is associated to the accumulation of CMV-specific memory T cells.^(^ ^15^ ^)^

### Proliferation

The existing *naïve* T cells increase through peripheral homeostatic proliferations to compensate the reduced thymic export in the aged. It was demonstrated that cytokine IL-7 plays a crucial role in the control of homeostatic proliferation of *naïve* TCD4^+^ and TCD8^+^cells.^(^ ^16^ ^)^ During aging, an increased number of memory T cells due to homeostatic proliferation is found; and unlike *naïve* T cells, they depend on cytokines IL-7 and IL-15.^(^ ^17^ ^)^ These findings suggested the increase in homeostatic cytokines during aging could also favor survival and expansion of memory T cells.^(^ ^17^ ^)^

Another change found in the elderly is the diminished *in vitro* proliferation capacity of T cells in response to different mitogens, as comparison to young individuals.^(^ ^18^ ^,^ ^19^ ^)^ It is believed that the reduction in IL-2 secretion and in expression of CD25 (alpha chain of IL-2 receptor) and CD28 molecules (which, after linking with the B7 molecule, induce transcription of IL-2 and of cytokine receptor IL-2/IL-2R) should significantly contribute to this decreased proliferation capacity of T cells observed in elderly individuals.^(^ ^20^ ^)^

Immunosenescence can also be attributed to a phenomenon known as T cell replicative senescence, which is a process through which normal somatic cells reach an irreversible stage of cell cycle after several replication cycles. We currently know that persistent viruses and tumor antigens are among the agents leading to replicative senescence of T cells *in vivo*. Senescent TCD8+ cells accumulate in seniors, and often present antigen specificity of CMV, which suggests this common and persistent infection could promote immune senescence, resulting in function and phenotype alterations in the T cell repertoire. Senescent T cells have also been identified in patients with certain types of cancer, autoimmune diseases and chronic infections, like HIV. These cells are characterized by loss of the CD28 costimulatory molecule, shortened telomeres, and the elevated production of pro-inflammatory cytokines.^(^ ^21^ ^)^

### Cytokines

The increase in pro-inflammatory cytokines and drop in anti-inflammatory cytokines are present in the aging process, and this condition is known as inflammaging.^(^ ^22^ ^)^ The changes in cytokine production may be related to T cell senescence. DNA damages are found in these cells, such as double-strand break, inefficient repair, and reduced telomerase activity. The responses resulting from chronic DNA damage include mainly the activation of DNA-PKcs, the catalytic subunit of DNA-dependent protein kinase. DNA-PKcs activity influences intracellular signal pathways through increased activity of NF-kB, which may contribute to the production of pro-inflammatory cytokines, such as IL-1, IL-6, and tumor necrosis factor-α (TNF-α), thus characterizing the inflammaging profile in elderly individuals.^(^ ^23^ ^)^ The increase in myeloid progenitor production by the bone marrow in the aged may also be associated to the inflammaging process, since monocytes of these individuals present elevated cytokine secretion under basal conditions, such as pro-inflammatory cytokines (IL-6, TNF-α) and low levels of anti-inflammatory cytokines (IL-10).^(^ ^24^ ^)^

Interleukin-2 secretion decreases as aging progresses. Hence there is reduced T cell proliferation capacity, drop in IL-2 synthesis and release (feedback), besides a decline in T cell capacity to express IL-2 receptor.^(^ ^25^ ^)^

Long-living individuals generally present systemic inflammation signs, decrease in antioxidants, and a hypercoagulability state characterized by higher plasma levels of important factors involved in hemostasis balance. However, these individuals avoid or delay the onset of chronic aging-related conditions, such as type 2 *diabetes mellitus*, cardiovascular diseases, and invasive cancer, which suggests the inflammatory and hypercoagulable states are compatible with health and longevity. Franceschi et al., developed the hypothesis that alterations in the immune system are properties of an immune remodeling that comes with aging, in which adaptation mechanisms may have evolved under selective pressure to optimize the maintenance and repair of organs, tissues or cells, thus enabling longevity for some individuals.^(^ ^26^ ^)^

## MYELOID-DERIVED SUPPRESSOR CELLS

The unbalance between production of myeloid and lymphoid progenitor cells during aging favors the myeloid lineage, and this trend may be associated to increase of MDSC in seniors. Myeloid-derived suppressor cells are heterogenous and have a population of myeloid cells (granulocytes, macrophages, and dendritic cells) that are at their initial stage of development and present immune suppression capacity.^(^ ^27^ ^)^

In the bone marrow of healthy individuals, the hematopoietic cells differentiate into common myeloid progenitor cells and then into immature myeloid cells. Under physiological conditions, immature myeloid cells migrate to different peripheral organs, where they differentiate into dendritic cells, macrophages or granulocytes. However, some factors produced in conditions like the microenvironment of cancer, several infectious diseases, sepsis, trauma, bone marrow transplant, and some autoimmune diseases promote the accumulation of immature myeloid cells in these sites, hinder their differentiation, and induce their activation. In this context, these cells are denominated MDSC. In healthy individuals, immature myeloid cells account for approximately 0.5% of peripheral blood mononuclear cells.^(^ ^28^ ^)^

Most studies showed the immunosuppressive functions of MDSC require direct contact with the cells, suggesting they act through both T cells surface receptors, such as TCR, and release of short-duration soluble mediators. The main mechanisms involved in the MDSC-mediated suppression of T-cell function occur by means of arginase (encoded by ARG1), induced nitric oxide synthase (iNOS, also known as NOS2), increased production of nitric oxide (NO) and reactive oxygen species (ROS).^(^ ^28^ ^)^

Myeloid-derived suppressor cells may play an important role in some of the main aging-related morbidities, such as infections, cancer, and autoimmune diseases. Verschoor et al., analyzed MDSC in peripheral blood mononuclear cells of adult (19 to 59 years), senior (61 to 76 years) and frail elderly individuals (67 to 99 years). They found a significant increase in MDSC in senior and frail elderly individuals in comparison to healthy young adults. Moreover, 23 elderly donors and frail elderly individuals with a history of cancer (breast, lung, prostate, skin and colon) presented significantly higher figures of MDSC, despite being in complete or partial remission.^(^ ^7^ ^)^

The function of MDSC is not clear, be it in the physiological state, *i.e*. healthy young individuals, or during aging process. The age-related rise in MDSC may contribute to increased incidence of the above-mentioned diseases, which are associated with aging or may be a protection factor, such as in autoimmune diseases. A more in-depth investigation about the functional consequences of MDSC accumulation in the peripheral blood would provide a prognosis for conditions related to age, and the MDSC could be used as biomarkers.^(^ ^7^ ^)^

## CONCLUSION

By understanding each of the mechanisms originated by remodeling of the immune system brought by aging, we could use the cells addressed in the present study (T cells and MDSC) as early and minimally invasive biomarkers for aging-related diseases. The aim is to minimize the limitations of immunosenescence and ensure better treatment for the vulnerable elderly population. Therefore, a deeper understanding of the immune system of long-living individuals could guide preventive and/or interventional policies.
